# Examining the Backpack Weight Relative to Students’ Body Weight Among Urban and Rural Schoolchildren: A Cross-Sectional Study

**DOI:** 10.7759/cureus.58861

**Published:** 2024-04-23

**Authors:** Nishath A Ahmed, Nida A Ahmed, Kailash Narendran, Afreen Shahid, Darshan k Raj, Nishank Kashyap, Amisha Palande, Gajalakshmi S., Prashanth A, Roshan Prasad, Gaurav Mittal

**Affiliations:** 1 Pediatrics, Dr. B.R. Ambedkar Medical College and Hospital, Bangalore, IND; 2 Trauma and Orthopaedics, Barnsley Hospital NHS Foundation Trust, Barnsley, GBR; 3 Surgery, East Lancashire Hospitals NHS Trust, East Lancashire, GBR; 4 General Medicine, Dr. B.R. Ambedkar Medical College and Hospital, Bangalore, IND; 5 Pediatrics, Sri Siddhartha Medical College and Hospital, Tumkur, IND; 6 Family Medicine, MGM Institute of Health Sciences, Navi Mumbai, IND; 7 Physiology, Terna Medical College, Mumbai, IND; 8 Community Medicine, Veer Chandra Singh Garhwali Government Medical Science and Research Institute, Srinagar, IND; 9 Physiology, Mahatma Gandhi Institute of Medical Sciences, Wardha, IND; 10 Medicine, Jawaharlal Nehru Medical College, Datta Meghe Institute of Higher Education and Research, Wardha, IND; 11 Internal Medicine, Mahatma Gandhi Institute of Medical Sciences, Wardha, IND; 12 Research and Development, Students Network Organization, Mumbai, IND

**Keywords:** private school, government school, student body weight, school students, heavy backpack

## Abstract

Background

Schoolbags or backpacks have been an essential part of the education system for a long time. However, a hefty backpack causes the child to arch the back excessively or bend their head and trunk forward to withstand the weight of the schoolbag. If the student carries the backpack on one shoulder, he/she bends to the opposite side to compensate for the extra weight, which may damage the shoulders and spine. Considering these factors, the main aim of this study was to investigate the percentage of backpack weight in proportion to the student’s body weight regarding the new guidelines among urban and rural schoolchildren aged 12 to 15 years in Bangalore, Karnataka.

Methodology

In southern India, over a year, a cross-sectional study was conducted with 500 students who voluntarily participated after providing written consent. They completed a questionnaire, underwent vital and anthropometric measurements, and had their weights measured, including the weights of their backpacks. Sample bags were inspected to determine contributing weight factors and evaluate adherence to timetables.

Results

The average weight carried by children of all ages was 6.53 kg, averaging 13.53% of their body weight. Among males, the percentage carrying backpacks weighing over 10% of their body weight was 80.9%, while among females, it was 85.7%. Of all the males carrying bags weighing more than 10% of their body weight, 67.7% attended government schools, while 32.3% attended private schools. Among females studying in government schools, 63.6% carried backpacks weighing more than 10% of their body weight, while among those in private schools, 36.4% carried bags exceeding that weight.

Conclusions

The study concluded that despite regulations being implemented on backpack weight for children, a significant number still carry bags exceeding 10% of their body weight among both urban and rural school children. This could elevate pressure on neck and back muscles, leading to excessive fatigue and potential damage to the skeletal system, ultimately contributing to spinal deformities.

## Introduction

The predominant instructional strategy that the Indian educational system supports is didactic lectures, which promote passive knowledge acquisition. Typically, school students carry an array of items to and from school each day, including textbooks, notebooks, lunch, snacks, sports/music equipment, sweaters, blazers, and personal items such as stationery. Collectively, these items contribute to a substantial weight load that children must bear for a significant duration throughout the school year.

Besides the obvious fact that the highly competitive nature of education in the present scenario is leading to mental strain, the physical implications of education are hugely underplayed. There has been concern from the Government of India, the Directorate of Education, comprising the school branch, educationists and medical professionals, parents, and children regarding schoolbags [1]. It is believed that heavy schoolbags may be a cause of musculoskeletal pain (MSP), particularly in the lower back and neck [2]. More specifically, the lack of availability of a locker facility at school appears to be of importance, which means that students must carry their belongings for longer durations, which may add to a student's risk of developing MSP.

The physical demands associated with carrying a schoolbag are influenced not only by the dry weight of the bag but also by factors such as the design of the bag, strap adjustment, duration and frequency of carriage, and how the weight is compensated. Additionally, the nutritional status of the child is likely to play a role in the overall impact of schoolbag carriage on their physical well-being. Primary school students tend to carry backpacks that are heavier compared to their body weight, in contrast to middle and high school students. However, it has been observed that students aged 13 years and older are at higher risk of chronic back pain as they also go through puberty and growth spurts.

Several studies have demonstrated that carrying heavy backpacks can notably affect the posture and gait of a child. Conversely, other research suggests that the excessive fatigue resulting from lugging heavy backpacks may be correlated with the occurrence of back pain. Furthermore, heavy schoolbags have been linked to subpar educational outcomes and increased absenteeism [2].

The growth patterns of the axial and appendicular skeletons are not synchronized. While spinal growth extends over a more prolonged period, the rate of growth of the appendicular skeleton is highest during puberty and starts to taper off around 18 years for boys and 16 years for girls. Ossification of the secondary centers occurs in the mid-twenties. The ideal alignment of the head, neck, and shoulder is characterized by the earlobe being in line with the acromion and the highest point of the iliac crest. This alignment creates a reference line that equally divides the body into dorsal and ventral halves [3].

Alterations in the alignment of the neck can lead to strain on cervical joints and result in an imbalance in the performance of associated muscles. Children are particularly susceptible to overuse injuries in this context. The cartilaginous component is significant as cartilage is a precursor to bone ossification. Different regions of cartilage, including articular cartilage at joint surfaces, epiphyses, and apophyses, are susceptible to distinct types of injuries. Articular cartilage is prone to damage from mechanical stress, while the epiphysis and apophysis are more susceptible to injuries arising from repetitive micro-trauma [4].

Undue pressure of the spine for a greater length of time due to heavy backpack carriage makes it susceptible to injury. To compensate for the posterior displacement of the center of gravity, there is a postural shift, that is, forward leaning at the hip, the inclination of the head, and rigidity of postural muscles [5].

There exists a correlation between the weight of the backpack and the occurrence of skeletal malformations like scoliosis or kyphosis. Carrying an excessively heavy backpack prompts the child to arch their back excessively or lean their head and trunk forward to endure the weight of the schoolbag. This places pressure on the neck and back muscles, leading to heightened fatigue and potential damage to the skeletal system, eventually contributing to spinal deformities [3-5]. In situations where the student carries the backpack on one shoulder, compensatory bending toward the opposite side occurs to counterbalance the extra weight. This practice may result in damage to the shoulders and spine [5].

Considering the role of backpack weight on the musculoskeletal health of children this study was carried out to scrutinize the percentage of backpack weight relative to the body weight of school-going children aged 12 to 15 years, in rural and urban areas of southern India.

## Materials and methods

Study design and setting

This study adopted a cross-sectional design spanning one year, from January 2021 to February 2022. The reporting and article preparation for the cross-sectional aspects of the study adhered to the Strengthening the Reporting of Observational Studies in Epidemiology (STROBE) recommendations. Data was collected by visiting three private (urban) and three government (rural) schools in South India. 

Selection criteria and study population

The sample size for the study was 500 students, from private schools and government schools. This was based on the prevalence [6], with a 10% error rate, a 5% significance level, and a 95% confidence interval. All participants in this study provided consent or waived it. The Institutional Ethics Committee, Dr. B.R. Ambedkar Medical College and Hospital, issued approval with reference number EC 40. Per the inclusion criteria, students in the eighth, ninth, and tenth grades were recruited for the study, irrespective of gender. Conversely, exclusion criteria were defined by several parameters, including students who do not provide consent, those with recent fractures or sprains, individuals diagnosed with musculoskeletal or neurological diseases, students with pre-existing spinal deformities, and those with any known physical handicap. Additionally, students with diagnosed systemic conditions such as diabetes mellitus, congenital heart disease, known renal disorders, and collagen vascular diseases were also excluded from participation in the study to avoid confounders.

Data sources and variables

After obtaining appropriate clearance from the school management, informed consent was secured, and students were asked to complete a questionnaire, which was also authenticated and approved by the institutional review board. Students were then requested to remove their shoes, and a digital weighing machine was utilized to measure their weight in kilograms, first with the backpack put on and then without it. To reduce observer bias, an individual who was neither involved in the study nor affiliated with the school supervised the weight recording process at various intervals as specified by the study protocol. The disparity between the two measurements provided the weight of the school backpacks. The backpacks were also inspected after confirmatory verbal consent from the participants. This was done to determine the backpack's weight factors, and adherence to the set timetable was assessed. Anthropometric measurements, encompassing height and weight per World Health Organization's (WHO) growth charts, were taken for schoolchildren.

Statistical analysis

Statistical data analysis was conducted using Microsoft Excel and IBM SPSS Statistics for Windows, Version 22.0 (IBM Corp., Armonk, NY). The collected data were presented as frequencies and percentages and then tested using Pearson's chi-square and Fischer's exact tests. Graphs were generated using Microsoft Excel and Microsoft Word. A *P*-value of <0.05 was considered statistically significant.

## Results

A total of 500 students of various backgrounds, belonging to government and private schools, participated in the study. It is essential to note that there were no substantial findings through the questionnaire that could be useful for the assessment of the objective of the study, nor did it prove to be confounding. As per the findings of the demographic data among males, 160 (32%) attend government schools, while 74 (14.4%) attend private schools. For females, 173 (34.6%) were enrolled in government schools, and 93 (18.6%) attended private schools. The total number of participants in the study is 500, providing a comprehensive overview of the gender distribution across different types of schools. Table 1 delineates the distribution of subjects by gender and type of school. 

**Table 1:** Distribution of subjects based on type of school and gender. Govt, government; Pvt, private

Gender	Type	Number	Percentage (%)
Male	Govt	160	32%
	Pvt	74	14.8%
Female	Govt	173	34.6%
	Pvt	93	18.6%
Total		500	100 %

**Figure 1:**
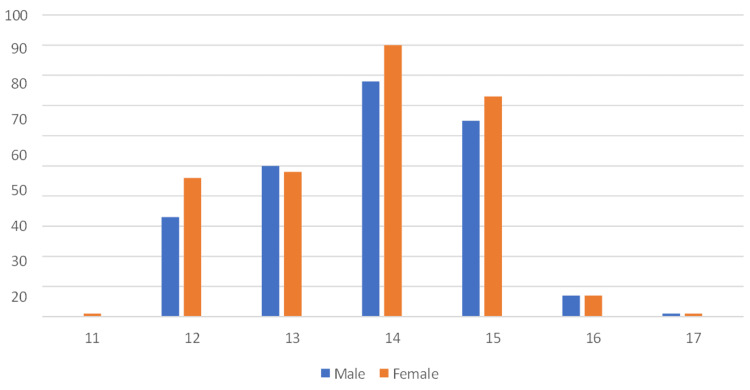
Clustered column showing age distribution among the school students. X-axis, age of students; Y-axis, number of students

Figure 1 illustrates the distribution of subjects by age and gender. Among 11-year-olds, there are no male participants, while one female participant constitutes 0.2%. At age 12, 33 males (6%) and 46 females (9.2%) are represented. The pattern continues with varying numbers for different age groups, and at age 17, there is one male (0.2%) and one female (0.2%) participant. Overall, the figure provides a breakdown of the study's subjects across different age brackets and genders. 

Table 2 presents the average weight of backpacks and their proportion to body weight based on age and gender. For 11-year-olds, females have an average backpack weight of 9 kg, constituting 18.75% of their body weight. At age 12, males carry backpacks averaging 5.72 kg (12% of body weight), while females carry 6.14 kg (12.58% of body weight). The pattern continues with variations for different age groups. Notably, at age 17, females carry backpacks averaging 9 kg, representing 17% of their body weight. 

**Table 2:** Mean weight of the backpack as compared to the percentage of participants' body weight. M, male; F, female

Age	Gender	Mean weight (kg)	Percentage of body weight (%)
11	F	9	18.75
12	M	5.72	12
	F	6.14	12.58
13	M	6.68	13.68
	F	6.06	12.88
14	M	6.23	13.1
	F	6.25	13.1
15	M	7.34	14.29
	F	7.32	15.07
16	M	6.57	12.6
	F	6	12
17	M	6	12
	F	9	17

Table 3 displays the distribution of backpack weights relative to the percentage of body weight and categorizes it by gender. Among males, 45 individuals (19.2%) carry backpacks representing less than 10% of their body weight, while 189 males (80.8%) bear loads exceeding 10%. In the female group, 38 individuals (14.3%) have backpacks weighing less than 10% of their body weight, while 228 females (85.7%) carry loads surpassing 10%. Overall, out of a total of 500 participants, 83 individuals (16.6%) have backpacks weighing less than 10% of their body weight, and 417 individuals (83.4%) carry loads exceeding 10%. The Pearson chi-square was calculated as 2.011 considering 1 degree of freedom and Fisher’s exact test score was 0.163. The *P*-value for the same came out to be 0.138, which was not significant.

**Table 3:** Backpack weight distribution based on gender. The Pearson chi-square was calculated as 2.011 considering 1 degree of freedom and Fisher’s exact test score was 0.163. The *P*-value for the same came out to be 0.138, which was not significant.

Backpack weight in terms of percentage body weight	Gender		Percentage		Total
	Male	Female	Male	Female	
Less than 10%	45	38	19.2%	14.3%	83 (16.6%)
Greater than 10%	189	228	80.8%	85.7%	417 (83.4%)
Total	234	266	100 %	100%	100%

## Discussion

The function of a bag is to aid a child in carrying books, stationery, and other educational articles such as sports instruments, musical organs, and lunch bags [7]. But this weight causes undue pressure on the developing spines of young schoolchildren. Back in 1997, the National Back Pain Association (UK) carried out a schoolbag survey and concluded that students should be authorized to transport no more than 10% of their body weight to school [8]. As per the researchers, "Typically, the weight carried by a student is unlikely to surpass 10-12% of their body weight," even though no specific supporting data were provided [9]. Voll and Klimt conducted a study measuring schoolbag weights, preferences of carriage, distance to school, mode of travel, and backpack habits in 1,522 school students spanning four classes (first to fourth grade). Half of the students reported their schoolbags as excessively heavy in a self-assessment questionnaire. Most children wore their schoolbags with both straps on their shoulders. The regional school council recommended that the weight of schoolbags should not surpass 11% to 13% of the student's body weight [10].

The results of this study were consistent with those of the study by Joshi et al. [11], which found that the average weight of backpacks in schoolchildren as 11-year-olds was 6.89 kg or 21.6% of body weight. The present study had one girl who carried a bag weighing 9 kg, which was 13.53% of her body weight. Twelve-year-olds had a bag weight averaging 7.78 kg, measuring 20.4% of their body weight, while in this study, boys carried a bag that averaged 5.72 kg and accounted for 12.58% of their body weight. Girls carried a relatively heavier bag, which weighed 6.14 kg and accounted for 12% of their body weight. In contrast to the present study, where boys carried bags weighing 6.68 kg, which was 13.68% of their body weight, and girls carried bags weighing 6.06 kg, which was 12.88% of their body weight, 13-year-old students carried an average weight of 8.05 kg, accounting for 18.9% of their body weight. The gross average weight reduced to 7.46 kg, accounting for 16.9% of body weight in 14-year-olds in the previous study, whereas in this study, it was almost the same for males and females, i.e., 6.25 kg, accounting for 13% of the average body weight.

In 2002, a study [12] was conducted in Italy, which indicated that children carried up to 22% of their body weight in a backpack, and 30% of children carried more than 30% at least once per week. Brackley and Stevenson, in 2004, were the first to come up with scientifically backed evidence for the maximal weight of backpacks for schoolchildren and recommended a schoolbag weight limit of 10-15 BW based on the findings of current literature [13]. The researchers suggest that parents should oversee the contents of their children's schoolbags daily, and schools should design timetables to minimize the need for children to carry multiple textbooks daily [14]. Additionally, reports of other backpack-related issues include functional scoliosis, rucksack palsy, and diminished lung functions [15].

The Children Schoolbags (Limitation on Weight) Bill, 2006 [16] stipulated that the weight of schoolbags should not exceed 10% of the body weight of the child carrying them, and students studying in kindergarten and nursery should not be allowed to carry any bags to school at all. Subsequently, various studies in different parts of India and similar studies abroad have revealed that schoolbags weigh up to 15% of the body weight, which is beyond permissible limits and is linked to postural instability [17-20]. As per the study by Ramprasad et al., the spinal posture changes with an increase in the weight of the backpack [21].

Until 2010, there was no regulation of the load carried by a child in the form of backpacks. Still, a study conducted in 2009 showed that the prevalence of back pain was 30%, whereas a study conducted in 2020 reported musculoskeletal pain in 77.2% of children [22]. The recommendation of a 10% cutoff for the maximum weight of a backpack relative to the student's body weight was proposed, accompanied by various practical methods to assist schools in attaining this goal for middle and high school students [23].

Despite regulations being implemented in 2010, it was found that children still carry a bag that weighs more than 10% of their body weight in a significant number. The purpose of implementing these regulations is to reduce stress on the spine, reduce the incidence of back pain, and reduce the unnecessary expenditure of energy of a child in carrying weight, as well as direct that energy to brain growth and overall development.

Limitations 

Along with these useful outcomes, there were a few limitations in the study, which can be addressed in future studies, giving wider scope and moving the field forward. One of the limitations was the comparatively small sample size, which covered only a few schools. Moreover, a wider range of age groups could have been addressed, considering that students carry backpacks right from kindergarten days or primary schooling. Interventions can be carried out to address the ongoing issues related to heavy backpacks. In-detail analysis of pain and other musculoskeletal can also be considered for future studies keeping track of the present results. 

## Conclusions

Heavy schoolbags can cause stress and fatigue among students. This study found that despite regulations on backpack weight for children, many students in both urban and rural school environments still carry bags that exceed 10% of their body weight. To address health issues stemming from this, it is recommended to establish new norms and rules to limit students from carrying schoolbags that surpass 10% of their body weight.
